# Interfacial assessment of cention forte vs. equia forte and two forms of calcium silicate cements at two time intervals

**DOI:** 10.1038/s41405-024-00252-1

**Published:** 2024-08-24

**Authors:** Heba Abdelkafy, Nada A. Salem, Rasha Mohamed Marzouk, Alaa M. Eldehna

**Affiliations:** 1 Department of Endodontic, Faculty of Dental Medicine for Girls, Al-Azhar University, Cairo, Egypt; 2 Department of Pediatric Dentistry and Dental Public Health - Faculty of Dentistry- October 6 University, Cairo, Egypt; 3 Dental Biomaterial Department, Faculty of Dental Medicine for Girls, Al-Azhar University, Cairo, Egypt; 4 Department of Pedodontics and Oral Health, Faculty of Dental Medicine for Girls, Al-Azhar University, Cairo, Egypt

**Keywords:** Pulp conservation, Non-bonded restorations

## Abstract

**Aim:**

Assessment of interfacial gaps and mechanical impact of the materials layering between Cention Forte and Equia Forte restorations with two forms of Calcium Silicate Cements (CSCs) at the interfacial surface at two-time intervals.

**Methodology:**

Six groups of 72 primary molars were categorized by restorative material type and CSCs: Cention Forte(C), Cention Forte without primer (Cx), and Equia Forte (EQ). All were applied over MTA Angelus powder (M) or Bio-C Repair putty (P). Restorative materials were applied immediately (subgroup A) or delayed (Subgroup B). SEM was used to detect interface gaps. EDX measured element migration from the interface at specific distances. Vickers Microhardness Tester assessed microhardness.

**Results:**

Regarding SEM, there were no gaps between CSCs interfaces of both types (Powder and Putty) with all restorations at two-time intervals. Microhardness, there was a statistically nonsignificant difference between subgroups A & B in all groups except at 200 µm in the Cention groups (subgroup A) was significantly lower than (subgroup B) (*P* = 0.002, 0.03) respectively. At 400 µm in the MTA Angelus powder Group Cx, subgroup A was significantly higher than subgroup B (*P* = 0.003*). While Bio-C Repair putty in Group EQ (subgroup A) was significantly higher than (Subgroup B) (*P* < 0.0001*).

**Conclusions:**

The delayed application of Cention Forte over two types of CSCs is useful in getting the maximum HV and, in turn, the long survival rate of the filling. Immediate application of Cention Forte without primer is better over both types of CSCs. The delayed application of Equia Forte over MTA angelus powder is more considerable.

## Introduction

One common restoration technique is layering dissimilar materials together. Coronal cavity base materials are used for pulp or tooth preservation in various procedures, such as MTA pulpotomies in primary and young permanent teeth and the presence of perforations of the pulp chamber floor. For instance, placing a permanent, well-sealed restoration is crucial to clinical success [1]. Base materials that have been used traditionally, such as zinc oxide-eugenol cement and calcium hydroxide, are not utilized as frequently as they once were. Calcium silicate cement is widely regarded as the preferred method of preserving pulp. It has favorable features such as biocompatibility, bioactivity, hydrophilicity, sealing ability, and low solubility. Furthermore, it demonstrated outstanding clinical results due to its ability to promote pulp tissue repair and encourage reparative dentin formation during the very early stages of pulp wound healing [2].

The setting reaction of Mineral Trioxide Aggregate (MTA) involves a hydration process where tricalcium silicate and dicalcium silicate react with water to form calcium hydroxide and calcium silicate hydrate gel. This reaction results in an alkaline PH, which is beneficial for dental applications as it promotes healing and tissue regeneration. Additionally, additives can be used to modify the properties of MTA, such as reducing the setting time and improving workability [3].

The MTA Angelus (Angelus, Londrina, PR, Brazil) demonstrates the benefit of a shorter period required for its initial hardening. The setting time is 15 min, whereas the newer Traditional ProRoot MTA takes approximately 2 to 3 h to set. Reducing the setting time is occasionally preferred by clinicians, as it allows them to confirm that the material has hardened at the time of placement. This enables them to proceed with restorative procedures without concerns about MTA washout. The decreased setting time of MTA Angelus is attributed to the reduction in the concentration of calcium sulfate, the compound responsible for the extended setting time in the original formulation [4].

Bio-C Repair (Angelus, Londrina, PR, Brazil) (BCR) is another new silicate-based hydraulic cement that is presented in a ready-for-use format. According to the manufacturer, the plasticizing material provides a higher plasticity, improve handling and insertion, which may facilitate use in clinical procedures. However, Bio-C Repair mainly was composed of carbon (34.81%), oxygen (34.51%), and Zirconium (13.83%), with a lower concentration of calcium compared to the other biomaterials. In addition, aluminum and silicon are trace elements [3, 4].

Several potential, influential factors impact the success rate of layering restoration with Calcium Silicate Cements (CSCs). Firstly, the interaction between CSCs and the overlying restoration leads to subsequent mechanical and physical property alterations. Secondly, the period elapsed between the placement of CSCs and the final restoration [1]. The final restoration applied over the CSC may vary based on availability and the dentist’s preference. Pre-prepared restorative capsules are deemed an innovative approach in dentistry due to their time-saving nature and easy application, which is particularly beneficial when treating children.

Recently, an innovative form of glass ionomer cement (GIC) called EQUIA Forte Fil (GC group) has been introduced. The reinforcement mechanism of this GIC relies on the existence of uniformly distributed ultrafine and highly reactive glass particles. Increasing the molecular weight of polyacrylic acid results in forming a new category of restorative glass ionomer cement (GIC) with exceptional mechanical characteristics. According to the manufacturer, this new GIC material is suitable for load-bearing class I, II and class V restorations. According to reports, this new GIC demonstrates enhanced flexural strength and durability against wear and acid erosion [5].

Cention Forte (Ivoclar Vivadent) - The new filling material belongs to the Alkasites material family, which provides high flexural strength and tooth-colored esthetics. The unique alkaline filler boosts the release of hydroxide ions to control pH levels during acid attacks, and demineralization can be prevented. The massive release of calcium and fluoride ions also provides a strong foundation for remineralizing dental enamel. This material has a high flexural strength because the polymers are highly cross-linked. The powder has different glass fillers, initiators, and pigments, while the liquid has dimethacrylates and initiators. The monomer matrix is mainly made up of UDMA. It has a moderate viscosity and strong mechanical properties [6].

This study aimed to evaluate the relationship and interaction between Cention Forte and Equia Forte final restorative materials with two forms of MTA at the interfacial surface at two-time intervals using a Scanning Electron Microscope (SEM) for quality assessment. The X-ray energy dispersive analysis (EDX) is used for quantity assessment. micro-hardness testing (mechanical impact of the layering of the materials).

The null hypothesis suggested that immediate contact of the evaluated CSCs with different restorative materials will not affect the interfacial properties of these materials.

## Materials and methods

The present is a randomized in-vitro study that was performed in the Pediatric, Endodontics, and Biomaterial Departments according to CRIS (Checklist for Reporting In-vitro Studies) guidelines and regulations.

Ethical approval from the Research Ethics Committee (REC), Faculty of Dental Medicine for girls, Al-Azhar University, Cairo – Egypt, was obtained with primary Code (P-PD-22-27) and final Code (REC-PD-23-19).

The sample size was calculated using a previous study [7] as a reference. According to this study, the minimally accepted sample size was 12 per group when the mean ± standard deviation of group I was 27.11 ± 7.24, while the estimated mean difference was 8.5 when the power was 80%. The type I error probability was 0.05. The *t*-test was performed using P.S. power3.1.6.

### Samples preparation and grouping

Seventy-two anonymous extracted primary molars were collected. The teeth were washed under running water, and any remaining soft tissue fragments were removed from their root surfaces and stored in a sterile saline solution. Coronal cavity preparation was done using #2 Endoaccess bur (Mani, Japan).

**Fig. 1:**
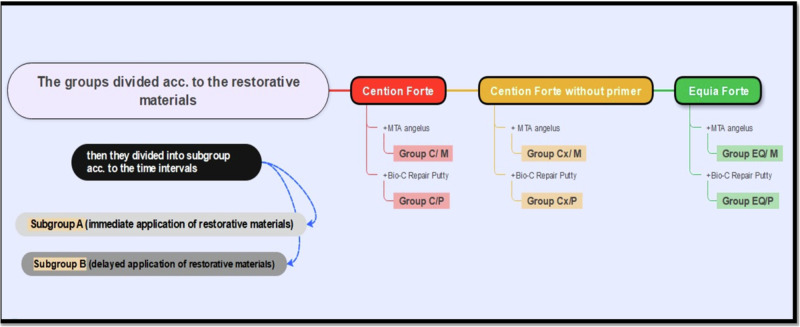
Samples Grouping Diagram.

After cleaning and drying the cavity, allocation sequence, randomization, and blinding were done. A random sequence was generated according to the CSC types and final restoration. using a random sequence generator website (https://www.random.org/sequences) into six experimental groups (Fig. 1). Allocation concealment was done by one clinician by inserting each sample in a separate envelope, followed by shuffling the envelopes and then writing a number from 1 to 72 on each envelope.

CSCs, either MTA Angelus- powder (M) and Bio-C Repair Putty (P), were prepared following the manufacturer’s instructions and then applied to coronal pulp champers over the canal orifices with a thickness of 3–4 mm using a wetted cotton pellet. These CSCs were then layered with **Equia Forte Fil** (EQ) and **Cention Forte(C)** (Ivoclar Vivadent) according to the assigned time interval, either immediate (subgroup A; *n* = 6) or delayed after the complete setting of the CSCs (subgroup B; *n* = 6).

- **Group C/ M (*n*** = **12):** Cention Forte and MTA angelus.
- **Group C/P (*n*** = **12):** Cention Forte and Bio-C Repair Putty.
- **Group Cx/ M (*n*** = **12):** Cention Forte without primer and MTA angelus.
- **Group Cx/P (*n*** = **12):** Cention Forte without primer and Bio-C Repair Putty.
- **Group EQ/ M (*n*** = **12):** Equia Forte and MTA angelus.
- **Group EQ/ P (*n*** = **12):** Equia Forte and Bio-C Repair Putty.

For subgroup A (immediate application), the assigned restorative materials were applied immediately before the CSCs setting as follows:

- Group C: A primer of Cention Forte restorative material was applied directly over the CSCs and cavity walls, then light-cured for 30 s. Then, the Cention Forte restorative material was applied over the cured primer.
- Group Cx: Cention Forte restorative material was applied directly over the CSCs without priming.
- Group EQ: Equia Forte restorative material was applied directly over the CSCs.

In contrast, in subgroup B, the restorations were not placed until two hours, ensuring that both types of MTA had been completely set.

### Sample preparation for evaluation

All specimens were stored in an incubator at 37 °C and 100% relative humidity for 1 week to ensure a complete setting of CSCs and mimic the internal body temperature. The samples with the layered materials were sectioned longitudinally and polished to expose the interface between the two materials in preparation for evaluation by SEM, EDX, and microhardness testing.

The samples were analyzed at the Grand Egyptian Museum using a scanning electron microscope (FEI Quanta 3D 200i Edx / thermofisher pathfinder). It was operated under low vacuum conditions for acceleration voltage 20.0 ~ 30.0kv using a large field detector with a working distance of 15 ~ 17 mm. Analysis of the interface was done using SEM at low magnification to detect the presence of possible marginal gaps. A rotation of the sample was made during observations to avoid artifacts related to overlapping margins. Gap areas larger than 5 microns in width and 20 microns in length that were visible at higher magnification (1000x) were deemed significant. While (EDX) was conducted at the interfacial surface, 200μ and 400μ distances to document elemental migration present at specific distances from the material interface.

For the Microhardness test, samples were placed in standard cylinders that were horizontally immersed in chemically cured acrylic resin (Acrostone, Cairo, Egypt) and modified for the hardness test such that the dentine surface was exposed to the Vickers indenter of the hardness testing device. The determination was made using a Digital Display Vickers Micro-hardness Tester (Model HVS-50, Laizhou Huayin Testing Instrument Co., Ltd. China) loaded with a Vickers diamond indenter and a 20X objective lens. A load of 50 g was applied to the surface of the specimens for 15 s. Three indentations were made on the surface of each specimen, evenly placed over a circle and at a minimum distance of 0.5 mm from the adjacent indentations. Using a built-in scaled microscope, the diagonal length of the indentations was measured, and the Vickers’ values were then converted into micro-hardness values.

The following equation was used to find the micro-hardness: **HV** = **1.85** **P/d2**

Where **HV** is Vickers hardness in Kgf/mm^2^, P is the load in Kgf, and **d** is the length of the diagonals in mm.

### Statistical analysis

The Statistical Package for Scientific Studies (SPSS 16®), Graph pad Prism, and Windows Excel were used for the statistical analysis. The given data was evaluated for normality using the Shapiro-Wilk and Kolmogorov-Smirnov test. The results showed that the significant level (*P*-value) was insignificant because *P*-value > 0.05. This meant that all the data came from a normal distribution (parametric data) that looked like a normal Bell curve. So, a Paired *t*-test was used to compare two different subgroups, and an independent *t*-test was used to compare two different HV. The One-Way ANOVA test was used to compare the groups, and Tukey’s Post hoc test was used for multiple comparisons.

## Results

### Characterization of uncontaminated materials (Figs. 2–4)

The X-ray energy dispersive analysis (EDX) of each material is shown in Tables 1, 2.

**Fig. 2:**
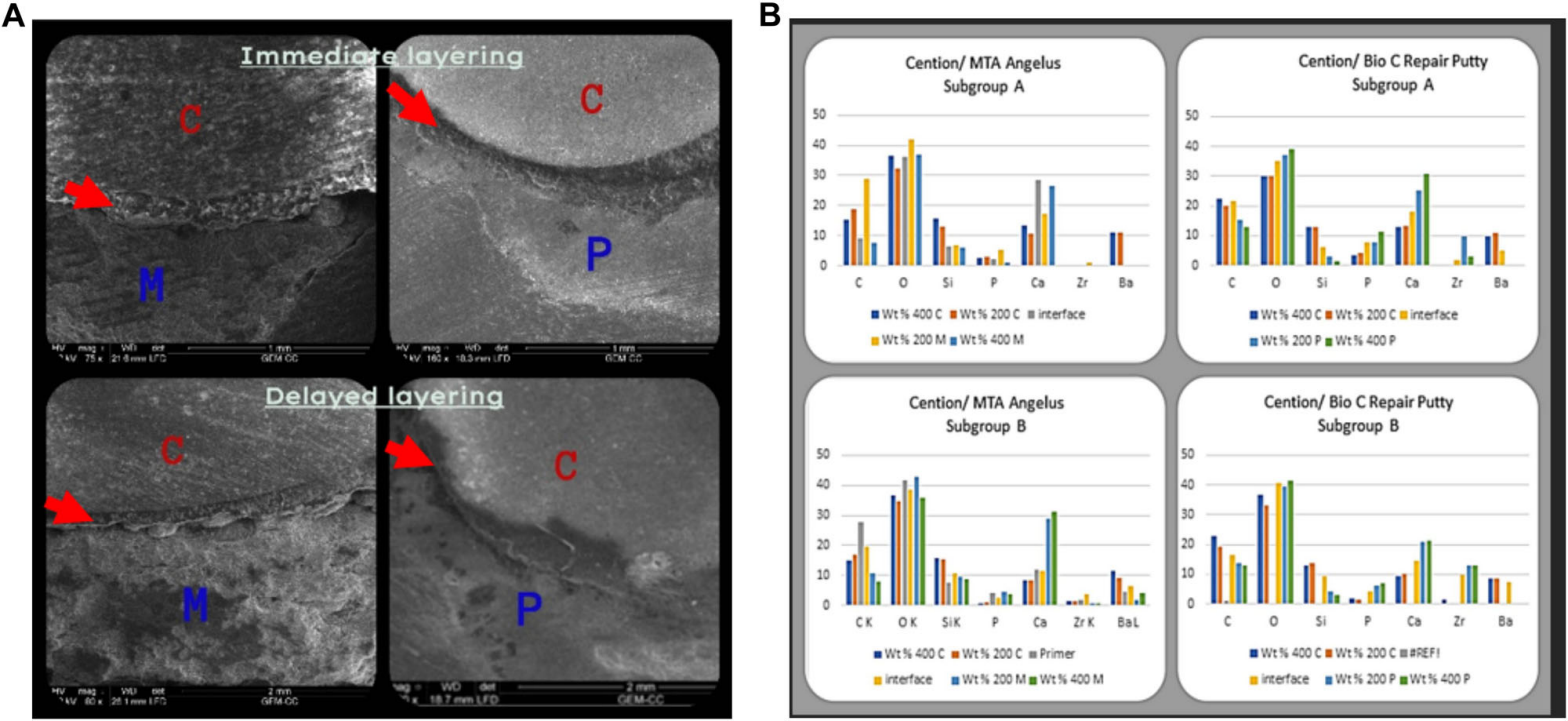
Interfacial regions of MTA Angelus (M) and Bio-C Putty (P) in contact with Cention Forte (C). **A** Backscatter electron microscope images show intimate contact. **B** EDX analysis of interfacial regions at 200 µ and 400 µ.

**Fig. 3:**
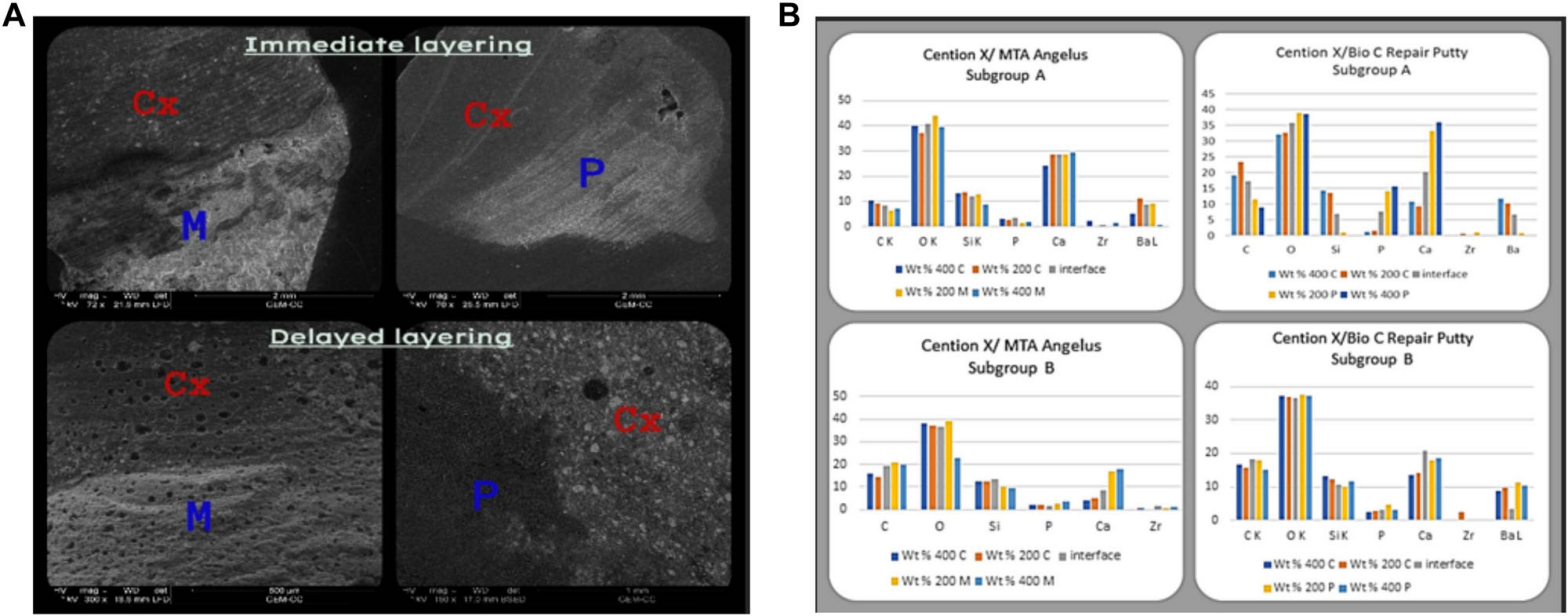
Interfacial regions of MTA Angelus (M) and Bio-C Putty (P) in contact with Cention Forte without primer layer (Cx). **A** Backscatter electron microscope images show intimate contact. **B** EDX analysis of interfacial regions at 200 µ and 400 µ.

**Fig. 4:**
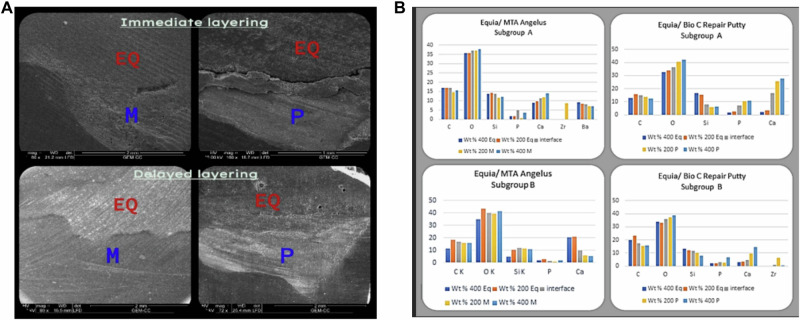
Interfacial regions of MTA Angelus (M) and Bio-C Putty (P) in contact with Equia Forte (EQ). **A** Backscatter electron microscope images show a minor gap in immediate layering. Conversely, delayed contact showed intimate contact. **B** EDX analysis of interfacial regions at 200 µ and 400 µ.

**Table 1:** EDX analysis of Equia Forte, Cention Forte with its Primer.

Equia Forte			Cention Forte			Primer		
Element	Wt %	At %	Element	Wt %	At %	Element	Wt %	At %
**O K**	43.76	58.97	**C K**	49.27	71.01	**C K**	63.34	73.84
**F K**	11.28	12.8	**O K**	14.97	16.19	**O K**	21.68	18.98
**NaK**	3.15	2.95	**F K**	1.79	1.64	**SiK**	10.77	5.37
**AlK**	12.42	9.92	**AlK**	2.47	1.59	**P K**	3.29	1.49
**SiK**	12.37	9.49	**SiK**	8.96	5.52	**K K**	0.92	0.33
**P K**	1.66	1.15	**CaK**	4.68	2.02	**C K**	63.34	73.84
**K K**	3.05	1.68	**BaL**	9.21	1.16	**O K**	21.68	18.98
**SrK**	12.31	3.03	**YbL**	8.64	0.86	**SiK**	10.77	5.37

**Table 2:** EDX analysis of MTA Angelus and Bio-C Repair.

MTA Angelus			Bio-C Repair		
Element	Wt %	At %	Element	Wt %	At %
**O K**	36.03	63.34	**O K**	30.34	57.37
**AlK**	1.87	1.95	**AlK**	1.17	1.32
**SiK**	8.33	8.34	**SiK**	7.6	8.19
**CaK**	33.06	23.2	**ZrL**	30.32	10.06
**W L**	20.72	3.17	**CaK**	30.57	23.08

Regarding restorative materials, Equia Forte analysis showed that it is composed mainly of Oxygen, fluoride, Aluminum, and Silicon with a lower percentage of Sodium, Potassium, and Phosphorus. Centione Forte contained a large percentage of carbon with less oxygen, fluoride, aluminum, and silicon than Equia Forte. In addition to Calcium and Barium. Moreover, its primer also contains Carbon, Oxygen, Silicon, Phosphorus, and trace elements of Potassium. On the other hand, MTA Angelus and Bio-C Repair specimens mainly consist of oxygen and calcium in addition to aluminum and silicon, while Bio-C Repair contains Zirconium in addition.

### Scanning electron microscopy and EDX of interfacial regions of CSCs and restorative materials (Figs. 2–4)

The data analysis revealed that there were no gaps at any of the interfaces between CSCs of both types (Powder and Putty) with the two restorative materials at two-time intervals. In subgroup A of group EQ/P, there was a minor gap in two samples, which was thought to be statistically insignificant. Figures 2 (A, B), 3 (A, B), and 4 (A, B).

### Microhardness comparison between different groups (Figs. 5, 6)

There was an insignificant difference between subgroups A and B in all groups except:

**Fig. 5:**
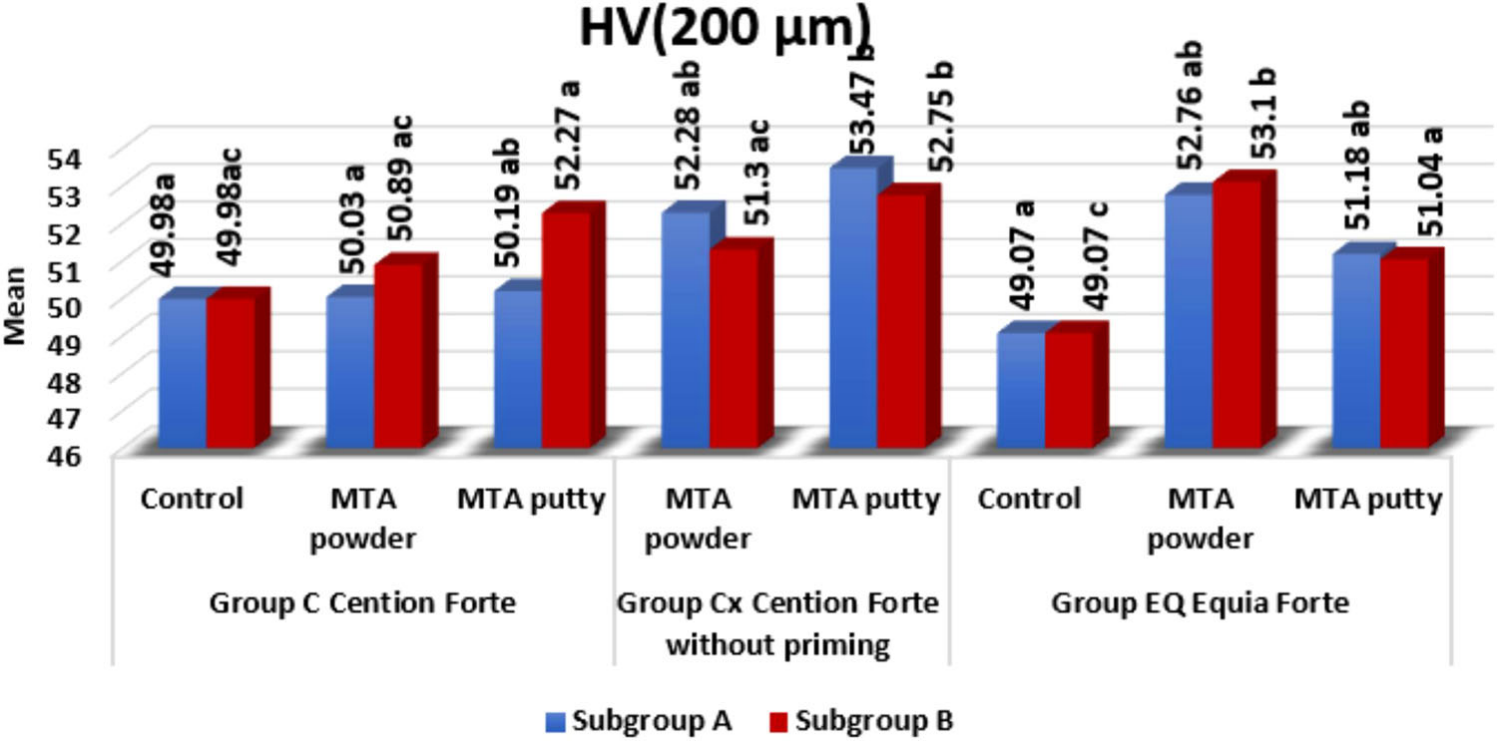
Bar chart showing subgroups A & B in all groups at 200 µ. Lowercase letters Means with the same superscript letters were insignificantly different as P > 0.05. Lowercase letters Means with different superscript letters were significantly different as P < 0.05.

**Fig. 6:**
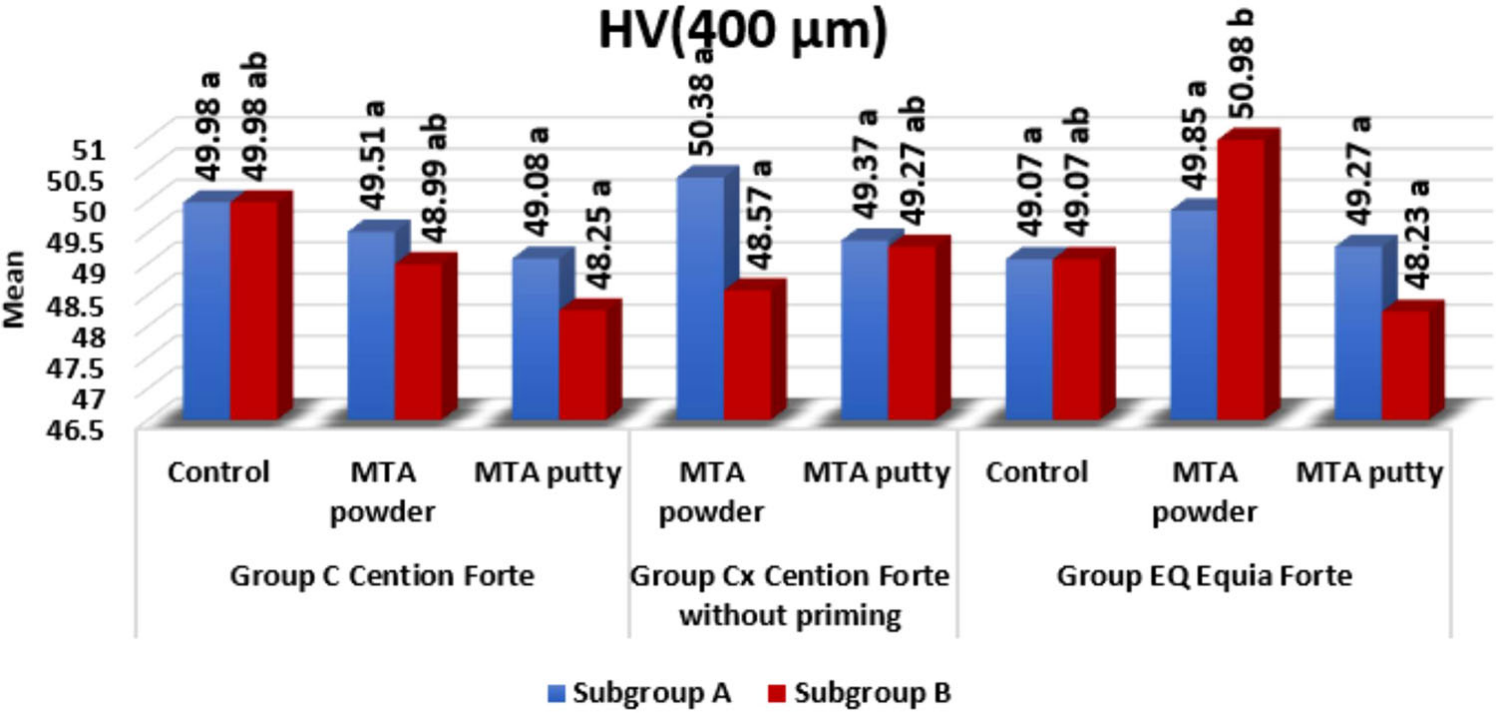
Bar chart showing subgroups A & B in all groups at 400 µm. Lowercase letters Means with the same superscript letters were insignificantly different as P > 0.05. Lowercase letters Means with different superscript letters were significantly different as P < 0.05.

### HV (200 µm)

In MTA Angelus powder and MTA Bio-C Repair putty, in group C (Cention Forte), subgroup A was significantly lower than subgroup B, as *P* = 0.002* and *P* = 0.03*, respectively.

### HV (400 µm)

MTA Angelus powder in Group Cx (Cention Forte without priming) as Subgroup A was significantly higher than Subgroup B as *P* = 0.003*. While Bio-C Repair putty in Group EQ (Equia Forte) as subgroup A was significantly higher than subgroup B as *P* < 0.0001*.

## Discussion

Achieving a complete seal is the major requirement for any restoration, especially if this restoration is used in combination with another one. In recent years, numerous restoration types have been introduced in dentistry, including Equia Forte and Cention Forte, in addition to the different types of calcium silicate cement. However, each material has unique qualities like setting time, color, and consistency. When these restorations come in contact with CSCs each type reacts differently, and this response is also influenced by time factor. This relationship between these restorations and the CSCs had little discussed in the evidence [8]. Regarding the Cention Forte, the manufacturer didn’t remember any instructions regarding layering on any other material. So, the authors are interested in testing both options of layering, one without primer and one with primer.

The primary goal of our study is to measure the micro-gap between the two CSCs types and two restorations using scanning electron microscopy at various time points to evaluate the quality of this layering. In Cention Forte with the primer group, it was observed that there were no gaps between the layers. They were laid out in separate layers since the primer prevents them from becoming completely interlaced and prevents the molecules of the different layers from fusing. (Fig. 2A). On the other hand, in Cention Forte without primer, there are no gaps and a complete blending between the layers at the interfacial surfaces when layered over both types of CSCs (Fig. 3A) at both time intervals. Considering that both materials are alkaline [9] and since they have similar elements like calcium, silica, phosphorus, and potassium (Tables 1 and 2), there may have always been a significant amount of ionic exchange between them.

About the Equia Forte, it minimally drifted away from the Bio-C Repair Putty when it was layered directly on it, but this gap wasn’t displayed when the layering was delayed. This minor separation may be declared by the withdrawal of water from the Bio-C Putty into the glass ionomer. The lack of water in the MTA resulted in the inhibition of complete hydration of the material [10]. In contrast, the Equia Fort and MTA Angelus completely blend at either time interval because the MTA Angelus takes about 15 min to set and requires little moisture (Fig. 4A).

Regarding the comparison of microhardness among different groups, the current study exclusively focused on conducting a microhardness assessment only on restorative materials. Since microhardness is a characteristic of the surface, the control group initially in this study consisted of restorative materials only (Equia Fort & Cention Forte). However, as they may be impacted by the underlying CSCs, it is preferable to measure microhardness at the 200 µm and 400 µm contact between the two materials.

The microhardness results **at level 200** **µm**, The HV at the interface between the Cention Forte and MTA angelus (**C/M group)** and Cention Forte and Bio-C Repair Putty **(C/P group)** the following was observed: firstly, no significant difference with the control group when Cention Forte came in contact with CSCs immediately, this may be attributed to that the setting reaction of MTA angelus is not completed and has not reached its final HV yet. In addition, the priming step, which contains self-etching material, will decrease the PH of the media, which will affect the setting reaction of the MTA angelus, which needs an alkaline environment to set [11]. Secondly, When the Cention Forte is applied over (MTA angelus, MTA putty) lately after complete setting, there is an increase of the HV, which is due to the complete setting of the materials, and final strength and hardness are reached. Where Cention Forte starts to release fluoride, calcium, and hydroxide ions, and the MTA forms silicate salts. Some unreacted silica particles act as a filler that increases strength and hardness [12].

At the same level but on the interface between the Cention Forte without primer and MTA angelus **(Cx/M group)** and the Cention Forte without primer and Bio-C Repair Putty **Cx/P group**, the following was observed. firstly, there is a significant increase in HV from the control group either with the immediate or delayed application of the Cention Forte without primer over MTA angelus or putty. The immediate application gives a higher HV than the delayed application. This may declare the interlocking that takes place between irregularities present on the surface of the freshly mixed MTA (powder or putty) and the freshly mixed Cention Forte without a barrier between the two materials at the interface. Moreover, the setting reaction of the Cention Forte is done as a polymerization reaction using a light curing device (hydrophobic material), so it will not uptake water from MTA [13]. Moreover, the HV of this group is higher than the HV of the previous one; this may be attributed to the absence of the primer step, which means that the pH of the environment is favorable for the setting of MTA without interfering with self-etching material.

Finding the HV where the Equia Forte and MTA angelus **(EQ/M group)** and Equia Forte and Bio-C Repair Putty **(EQ/P group)** interface, on the one hand, there is a significant increase in HV from the control group either with the immediate or delayed application of the Equia Forte over MTA angelus or putty. The HV of delayed application of the restorative material on MTA angelus powder is slightly more than the HV of immediate application due to the synthesis of silicate salts, hydroxyapatite, and fluoro-apatite at the interface, which greatly increases hardness (Fig. 5). This finding was explained by Martin RI and Brown PW [14]^,^ who determined the entire stoichiometric hydroxyapatite reaction that formed in an aqueous solution by acid-base reactions involving CaHPO4 and Ca4(PO4)2 O that happened in delayed time.

On the other hand, MTA angelus powder gives higher HV than MTA putty in both immediate and delayed applications of Equia Forte. This may be related to the rheological properties (viscosity, consistency) of the MTA supply form. The high water content of MTA angelus allows easier in and out movement of ions from both materials at the interface [15].

**When it comes to level 400** **µm**, The HV at the interface between the Cention Forte and MTA angelus (**C/M group) and** Cention Forte and Bio-C Repair Putty **(C/P group)**, there is a slight decrease in HV of delayed subgroups, either MTA angelus powder or putty. This may be due to the migration of elements from the deeper level to the most superficial level as the setting reaction proceeds (Fig. 2B). About the HV at the interface between the Cention Forte without primer and MTA angelus **(Cx/M group) and** Cention Forte without primer and Bio-C Repair Putty **(Cx/P group)**, the immediate application over MTA angelus powder is markedly higher than the delayed application as both materials in contact are still fresh and can interact with each other through ion exchange and form stronger mechanical interlocking on a deep level.

Subsequently, on the same level, the HV at the interface between the Equia Forte and MTA angelus **(EQ/M group)** and Equia Forte and Bio-C Repair Putty **(EQ/P group)**, firstly, the highest HV is achieved when delayed application of Equia Forte over fully set MTA angelus powder takes place. It is asserted that both Equia Forte and MTA angelus powder are hydrophilic in nature [16, 17]; thus, immediate insertion of the restoration over MTA angelus powder will lead to water uptake by the restoration from MTA that will affect its setting reaction inversely and may cause cracks on the surface decreasing the surface hardness. In the case of Bio-C Repair Putty, the immediate application of Equia Forte is higher than the delayed one but still lower than the HV of MTA angelus powder. This may be because it doesn’t need the same amount of water to complete its setting as the powder type, so the surface will not be affected or cracked when it comes in contact immediately with the restorative material.

Within the limitation of this study, it could be concluded that:

- When using Cention Forte as a final esthetic restoration, the delayed application is useful either over MTA angelus powder or Bio-C Repair Putty to get the maximum HV and, in turn, the long survival rate of the filling.
- When using Cention Forte, it is important to apply the primer only to the walls of the tooth structure, being careful not to let it come into contact with the MTA.
- When using Cention Forte without primer as a final esthetic restoration, use it immediately over either MTA angelus or Bio-C Repair putty to get the highest HV. However, the tooth restoration interface may be subjected to microleakage due to the absence of primer, as it helps to overcome the hydrophobicity of the material (no intimate contact will occur).
- When using Equia Forte as a final filling material, delayed application over MTA angelus powder will give the optimum condition.

### Why this paper is important to pediatric dentists

This research may guide in cases of MTA pulpotomy in both deciduous and early permanent teeth as follows:

- A deep understanding of the interaction between various layered materials.
- A knowledge of how this layering affects microhardness and filling longevity.
- The best timing for layering the final mentioned restorations over the different types of CSCs.

### Supplementary information

SI Table 1

## Data Availability

The data is available with the corresponding author if needed.
