# Supplementary material for: Interfacial assessment of cention forte vs. equia forte and two forms of calcium silicate cements at two time intervals
Source: BDJ Open. 2024 Aug 24;10:68. doi: 10.1038/s41405-024-00252-1 (PMC11344803; doi:10.1038/s41405-024-00252-1)
Supplement: Supplementary file 1 — SI Table 1 [file 41405_2024_252_MOESM1_ESM.pdf]

SI Table 1: Mean and standard deviation of subgroup A & B in all groups at 200 µm and 400 µm, comparison between subgroups, comparison between MTA powder and MTA putty:

			Subgroup A (Immediate)		Subgroup B (after 2hours)		P value
			M	SD	M	SD	
HV(200 µm)	Group I Cention Forte	Control	49.98 [a]	1.00	49.98 [ac]	1.00	1.00 ns
		MTA powder	50.03 [a]	1.43	50.89 [ac]	1.41	0.002*
		MTA putty	50.19 [ab]	3.30	52.27 [a]	1.43	0.03*
	Group II Cention Forte without priming	MTA powder	52.28 [ab]	1.64	51.30 [ac]	1.24	0.21 ns
		MTA putty	53.47 [b]	1.63	52.75 [b]	1.62	0.42 ns
	Group III Equia Forte	Control	49.07 [a]	0.98	49.07 [c]	0.98	1.00 ns
		MTA powder	52.76 [ab]	1.66	53.10 [b]	1.06	0.71 ns
		MTA putty	51.18 [ab]	1.93	51.04 [a]	1.56	0.82 ns
	P value		0.001*		<0.0001*		
HV(400 µm)	Group I Cention Forte	Control	49.98 [a]	1.00	49.98 [ab]	1.00	1.000 ns
		MTA powder	49.51 [a]	1.34	48.99 [ab]	0.99	0.21 ns
		MTA putty	49.08 [a]	2.80	48.25 [a]	1.23	0.21 ns
	Group II Cention Forte without priming	MTA powder	50.38 [a]	2.15	48.57 [a]	1.34	0.003*
		MTA putty	49.37 [a]	2.15	49.27 [ab]	1.43	0.85 ns
	Group III Equia Forte	Control	49.07 [a]	0.98	49.07 [ab]	0.98	1.000 ns
		MTA powder	49.85 [a]	1.40	50.98 [b]	1.01	0.07 ns
		MTA putty	49.27 [a]	1.02	48.23 [a]	1.12	<0.0001*
	P value		0.87 ns		0.002*		

M: mean   SD: standard deviation
*Ns: non-significant difference as P>0.05   *Significant difference as <0.05.*
Means with the same superscript letters were insignificantly different as P>0.05.
Means with different superscript letters were significantly different as P<0.05.
